# How Do Contexts Affect Physicians' Clinical Reasoning? A Narrative Review

**DOI:** 10.15694/mep.2020.000032.1

**Published:** 2020-02-14

**Authors:** Alice Mason, Rachel Locke, Rosie M Lusznat, Colin Coles, Mike G Masding

**Affiliations:** 1 Royal Hampshire County Hospital; 2 University of Winchester; 3 Health Education England (Wessex)

**Keywords:** Clinical reasoning, context

## Abstract

This article was migrated. The article was marked as recommended.

Background

Research about clinical reasoning has tended to focus on the individual, assessing their ability to perform clinical reasoning tasks. However, recent studies have noted that clinical reasoning varies with the clinical context.

Objectives

The purpose of this narrative review is to examine how the context can affect physicians clinical reasoning skills.

Methods

A narrative literature review was conducted by searching PubMed, PsycINFO and Embase via Ovid using the search terms clinical OR critical AND thinking OR judgement OR reasoning. Of 22,296 results found, 25 studies were found to be relevant to our review.

Results

Most studies focused on diagnostic skills. Contexts affecting clinical reasoning fell into three broad categories: patient, physician and environmental (the physical and social setting) factors. Patient contexts researched included factors both personal to the patient and their physical disease manifestations. Physician contexts included experience, age, exposure to similar diagnoses, incorrect diagnostic suggestion, emotions, and the use of reflection and checklists. Environmental contexts included time pressure, unfamiliarity with surroundings, dealing with uncertainty and high-stakes outcomes. The effect of applying more than one contextual factor increasing cognitive load, was explored.

Conclusion

This original review suggests that the context can affect a physician’s clinical reasoning abilities. This review identifies areas for continued research, including which contexts have a negative or positive impact, and the effect of multiple contexts (cognitive loading) on clinical reasoning. Further empirical research is needed to investigate these areas in more depth and to establish how far these benefits have an impact in practice.

## Introduction

Clinical reasoning (CR) has been defined as a way of thinking and decision making in professional practice ( Higgs and Jones, 2000), requiring physicians to sort through a cluster of features and accurately assign a diagnosis and develop a treatment strategy ( Eva K, 2005). The context in medical education has previously been defined as a complex system evolving over time, with the outcome being driven by interactions and feedback among patient, physician, setting, and their interactions ( Durning *et al*., 2010). ‘Context specificity’, the phenomenon of a physician’s performance varying on a case by case or situation to situation basis, has been well described ( Eva, 2003).

CR is important: diagnostic error contributes significantly to medical errors ( Kuhn, 2002), and complaints to governing bodies often concern clinical competence ( Gmcuk.org, 2019). A medical remediation programme showed that one third of referrals were due to CR deficits ( Guerrasio and Aagaard, 2014). Many errors are not solely due to lack of knowledge but also due to difficulties in physicians’ thinking in the real-world setting ( Kohn, 2009).

Research supports the view that CR skills can be developed and improved ( Scott, 2009; Thompson, 2004). Enhancing the way CR is performed by physicians in training has the potential to both reduce clinical errors and aid the transition from novice to expert.

This review aims to aid physicians and educators in recognising the effect of context on CR. It also suggests that this is an exciting area for research to build upon the existing literature and further examine how we might improve CR, thereby minimising clinical error, and how educators can help physicians in training enhance their CR.

## Methods

We conducted a narrative literature review searching key databases (PubMed, Psycinfo, EMBASE via Ovid) for peer reviewed empirical research articles in English published from January 2010 - September 2017. The search terms ‘clinical OR critical AND reasoning OR judgement OR thinking’ were used. Titles and abstracts were reviewed by the first author and a practising psychiatrist. Reference and citation searches yielded additional results. Any differences in opinion were discussed and resolved.

## Results

**Figure 1.**
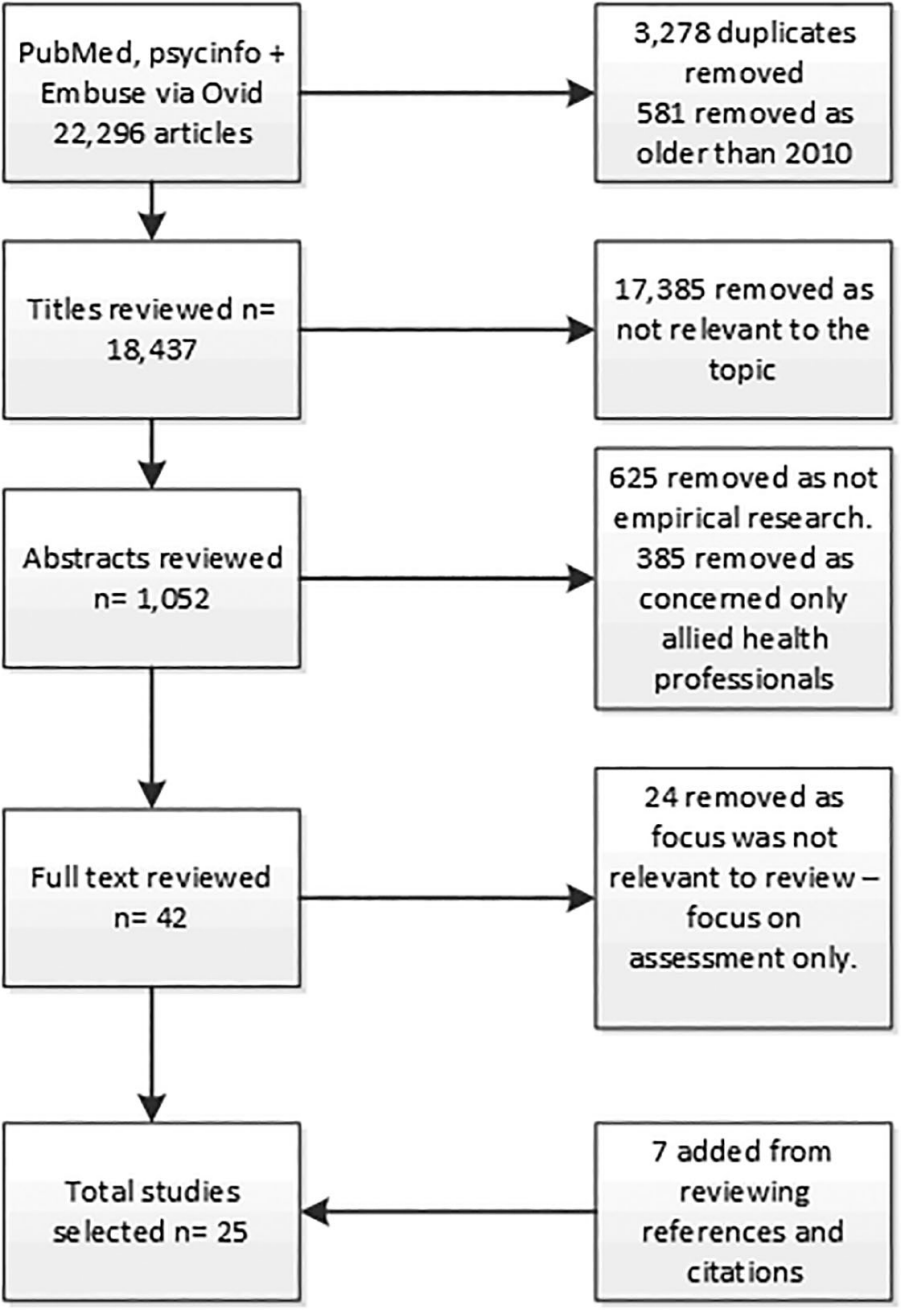
Search strategy for this review

A total of 22,296 articles were retrieved, reduced to once duplicates and articles older than 2010 were removed. A further 17,385 were removed as they were not relevant, 625 studies were removed as they were not empirical research, and 385 papers were removed as the studies were with allied health professions. Twenty-four studies were removed as they focused on the assessment of CR. An additional seven studies were added from reviewing references and citations. Twenty-five studies remained. Our search strategy is summarised in Figure 1.

**Figure 2.**
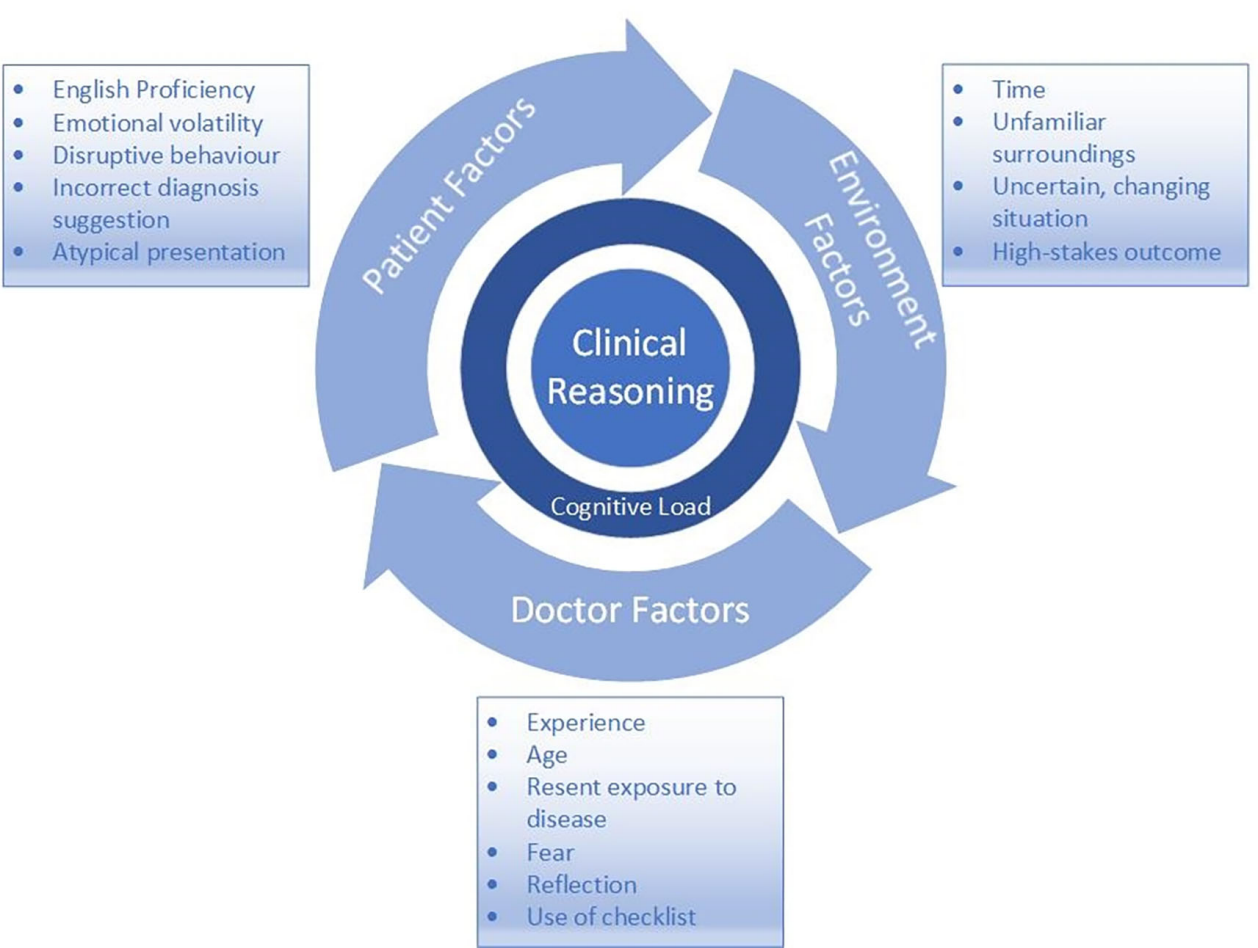
Contexts which affect CR

Of the 25 studies analysed, the contexts varied fell into three broad categories: patient factors, physician factors and environmental factors. Contexts varied in the studies which were shown to affect CR are outlined in Figure 2.

### Patient Factors

Eight studies varied patient factors. One study showed 25 physicians videotaped encounters. They altered one or more contextual factor: low English proficiency, emotional volatility, incorrect diagnosis suggestion or atypical presentation. Physicians thought aloud and wrote their diagnosis, management plan and the effect contextual factors had on their CR processes. Participants discussed the need for additional resources due to the contextual factor. Some participants dismissed the contextual factor whilst other viewed it as potentially beneficial - incorrect suggestion and emotional volatility were most likely to be dismissed, the presence of more than one factor was always viewed negatively. Participants were more likely to miss key data if two contextual factors were present. The research team postulated that multiple contextual factors led to increased cognitive load, leading to a negative perception of the situation and missed key data ( Durning *et al*., 2011).

McBee *et al*. showed ten physicians videotaped encounters and asked them to think aloud about their CR. Videos had a contextual factor altered - low English proficiency, emotional volatility or both. In the presence of both contextual factors, participants experienced higher levels of diagnostic uncertainty and the desire for further information; including additional tests ( McBee *et al*., 2015). A follow on study asked 14 physicians to watch videotaped encounters. Each had one or more contextual factor altered (low English proficiency, emotional volatility or both). A higher cognitive load was not consistently associated with a poorer performance ( Ratcliffe *et al*., 2017).

Another study looked at patient’s disruptive behaviour and diagnostic difficulty of presentation. In case vignettes, patients were depicted as difficult (displaying distressing behaviours) or neutral. Case vignettes were deemed diagnostically difficult or simple. Sixty-three physicians evaluated the cases. Diagnostic accuracy was significantly lower for both the difficult patients (p=0.017) and the diagnostically difficult cases (p<0.001). Participants then reflected on the cases. Reflection improved diagnostic rates (p=0.002) ( Schmidt *et al*., 2016). The researchers repeated the study, this time investigating why difficult patients reduced diagnostic accuracy. They gave 74 physicians case vignettes, half with ‘difficult patients’ and half with ‘neutral patients’. Diagnostic scores were significantly lower for difficult patients (p < 0.01) and participants recalled fewer clinical findings and more behaviour observations from the difficult patients (p < 0.001) ( Mamede *et al*., 2016).

One study looked at patient appearance and the effect on CR. Forty-six physicians where given case-based scenarios with a patient picture. Investigators classed patient pictures as ‘poor and dirty’ in appearance or as ‘rich and clean’. There was no significant difference in diagnostic accuracy, however participants reported processing the case more extensively if the patient appeared ‘rich and clean’ (p = 0.04) ( Mohamed *et al*., 2016).

A study looked at the influence of salient distracting features, a feature strongly associated with a certain disease which may catch a physician’s attention triggering pattern recognition. They gave 72 physicians case vignettes; some had a salient distracting feature at the beginning, some at the end and some had none. Half the cases were deemed simple and half complex. For the complex cases a distracting salient feature at the beginning of the case reduced diagnostic accuracy (p < 0.001). There was no significant difference when the distracting salient feature was at the end ( Mamede *et al*., 2014).

A study looked at the bias effect of clinical history on physical examination. They gave 180 physicians a clinical history and asked for a diagnosis. Participants were then randomised into three groups. The first group were given physical findings which fit with the likely diagnosis from the history, the second group were given the physical findings of an alternative diagnosis with indistinct physical findings, the third were given physical findings of an alternative diagnosis with distinct physical findings. Physicians showed a significant decrease in diagnostic accuracy when faced with discordant physical findings (p < 0.0001) ( Sibbald and Cavalcanti, 2011).

### Physician

Ten studies varied physician factors. One study assessed availability bias. Researchers asked 18 first-year and 18 second-year physicians to evaluate six written clinical cases. Then participants diagnosed eight further clinical cases as quickly as possible aiming to induce non-analytical reasoning. Four of these clinical cases had similar clinical manifestations to the initial cases, but different diagnosis. They found that second-year physicians, who had been exposed to cases with similar clinical manifestations but different diagnosis in round one, were more likely to give the incorrect diagnosis (p=0.03). There was no significant difference for the first-year physicians. Finally, participants evaluated a further four cases, with two of the cases having similar clinical manifestations to previous cases, but different diagnosis. The participants were given a structure to follow, aiming to induce reflection. This improved correct diagnosis rates compared with nonanalytic reasoning for all participants (p=0.006) ( Mamede *et al*., 2010).

Recent media exposure to a disease and the effect on a physician’s diagnostic skills was analysed in one study. Investigators asked 38 physicians to read a Wikipedia entry about a rare condition. Six hours later participants evaluated eight written clinical cases. Two cases had similar clinical manifestations to the Wikipedia entry they had read. Participants were more likely to give the incorrect diagnosis if the case described was like the Wikipedia diagnosis they had read earlier (p=0.16). The participants then evaluated four of the cases again in a structured format. This subsequent reflection increased diagnostic accuracy (p=0.27) ( Schmidt *et al*., 2014).

A study looking at the influence of diagnostic suggestion asked 24 physicians to evaluate case vignettes, half with a correct diagnostic suggestion and half with an incorrect diagnostic suggestion. Participants were able to accept the correct diagnosis more easily than rejecting the incorrect diagnosis (p < 0.05) ( van den Berge *et al*., 2012a). In a similar study 38 physicians evaluated case vignettes, half had a correct diagnosis suggestion and half had an incorrect diagnosis suggestion. In this study, diagnostic performance did not differ between cases( van den Berge *et al*., 2012b).

A study investigated the effect of physician age on diagnostic accuracy. Eighty-nine physicians were split into age bands: under 30, 30-39, 40-49 and over 50. They worked through case vignettes and gave a diagnosis. Participants younger than 30 performed better than participants aged over 40 (p<0.01) St-Onge *et al*., 2015). One study looked at management plans of physicians and medical students. The study asked 20 fourth-year and sixth-year medical students and ten physicians to review written clinical cases and provide management plans. The physicians provided more accurate management plans as compared to the medical students, the format of the management plans of the sixth-year medical students were more like the physicians as compared to the fourth-year medical students ( Monajemi, Schmidt and Rikers, 2012).

Another study asked 191 physicians to use a checklist and assessed the effect on diagnostic accuracy. The participants performed a cardiology examination on a simulator patient. Half of participants then completed a checklist of the different components of the cardiology exam and repeated the examination. Completion of the checklist improved diagnostic accuracy (p=0.04) ( Sibbald *et al*., 2013). Another study looked at the use of a mnemonic TWED (T = threat, W = what else, E = evidence and D = dispositional factors) created to facilitate metacognition. Forty medical students were split into two groups, with the intervention group using the mnemonic. Both groups’ clinical decision making was assessed based on their performance in case-based scenarios. The intervention groups’ mean score was significantly higher as compared to the control group (p < 0.001) ( Chew, Durning and van Merriënboer, 2016).

### Environment

Eight studies looked at the environment and CR. A study retrospectively interviewed 21 general practitioners working out of hours. They described two recent cases and the influences on their CR were explored. Participants perceived out of hours practice differently, they referred to ‘firefighting’: working in unfamiliar surroundings under time pressure. They dealt with the immediate situation, then stopped collecting information and ensured there was reliable safety netting. They also reported a lack of feedback on which to reflect and learn ( Balla *et al*., 2012).

Another study sought to assess the effect of time pressure on diagnostic accuracy. They gave 42 physicians case vignettes. The intervention group were manipulated to make them feel that they were under time pressure and falling behind. The control group received no such manipulation. The mean response time for the intervention group was significantly quicker, but with significantly lower diagnostic accuracy ( ALQahtani *et al*., 2016). However, two other studies did not mirror this result. One study recruited 46 emergency medicine physicians and 152 less experienced physicians. They were given case vignettes; the first ‘slow conditions’ group were encouraged to give due consideration to each case, the second ‘fast conditions’ group were asked to imagine they were in an emergency department working quickly and were given less time. Diagnostic accuracy was significantly higher for the emergency physicians compared to the less experienced physicians (p<0.0001). However, although the ‘faster conditions’ group did respond quicker than the ‘slow conditions’ group there was no significant difference in diagnostic accuracy ( Monteiro, 2015). In a similar study 204 physicians were split into the ‘speed cohort’ and the ‘reflect cohort’. They were given case vignettes, the ‘speed cohort’ were encouraged to work quickly and accurately and had a visible count down timer. The ‘reflect cohort’ were encouraged to be thorough, consider all data and had no count down timer. There was no significant difference in the diagnostic accuracy, although the reflect group were significantly slower (p<0.0001) ( Norman *et al*., 2014). A separate study also limited participants’ time. Twenty-five physicians were shown videotaped encounters. Participants thought aloud and completed a post encounter form. The time given to complete the post encounter form was randomly varied. During the ‘think aloud’ process researchers measured the cognitive load on the participant. Increased cognitive load and reduced time to complete the post encounter form had a negative impact on performance ( Durning *et al*., 2011).

Three studies looked across the board at patient, physician and environmental factors. Forty consultant dermatologists were interviewed regarding what influenced their decision to discharge patients from clinic. The list of influences was extensive; of the patient-based influences most participants highlighted the disease type and the patient’s ability to cope with their disease. Of the physician-based influences, most physicians highlighted their own amount of experience and their confidence in the decision. Of environment-based influences, physicians felt a supportive, well run department and confidence in local primary care services facilitated earlier discharge. Pressure from hospital managers also influenced their decision ( Harun *et al*., 2015). Another study interviewed ten infectious disease experts asking them to recall a complex antibiotic-prescribing incident. They focused on what made the incident complex. Several themes emerged; the overall clinical picture not matching the clinical pattern - an unexpected outcome, risky patient characteristics and unusual cases. A lack of comprehension of the situation - lack of or conflicting data, lack of evidence for treatment effectiveness, no diagnosis and gaps in the physician’s knowledge. Participants also highlighted the effect of social and emotional pressures - frustration and regret, liability or fear and the pressure and potential conflict of multiple care providers ( Islam *et al*., 2015). In another study investigators interviewed 36 newly qualified physicians, asking what influenced their behaviour when identifying and managing acutely deteriorating patients. They described difficulties translating theoretical knowledge into practice and making decisions in uncertain circumstances. They referred to the fear of being wrong or causing harm. They described the strong presence of hierarchy deterring new physicians from asking for help, fearing they would fall short of their senior’s expectations. They also described the pressure of the acutely unwell patient; time pressure, high-stakes outcome, heavy information load and a changing situation; causing them to freeze and having an emotionally negative impact ( Tallentire *et al*., 2011).

## Limitations

Limitations of the study include difficulty in measuring CR, with most studies using diagnosis as a surrogate marker. Most studies were done with a small number of participants, often with inexperienced clinicians and, so it may not be possible to draw broad conclusions based on this group. Few studies were done in the real-world clinical setting, but rather with paper, online or simulated cases, this limits the validity of conclusions regarding the effect of context. There are a very small number of investigators doing this work which could be a potential source of bias. Many of the studies depended on participant self-reporting of the effects of context on CR, physicians’ own understanding of context on their CR may be limited. The search was restricted to medical physicians only, much work has been done within other fields, particularly nursing. Work other fields could provide useful insights which could help inform further research into physicians’ CR. None of the trials looked at long-term follow up data, and most of the studies were performed by a small number of research groups. This paper is a narrative review and therefore open to selection and interpretation bias.

## Discussion

Contextual factors in CR which have been researched in the literature fall into the categories of patient, physician and environment. Studies have used various methodologies including case vignettes, videotaped encounters, simulation and interviews. Interventional studies mainly used correct diagnosis as an outcome measure, some studies looked at management and physician reflections.

Several patient factors, both personal to the patient and physical disease manifestations, appear to have an impact on a physician’s CR. Some studies demonstrated that altering patient factors, including low English proficiency, an incorrect diagnostic suggestion, emotional volatility and disruptive behaviour can lead to poorer physician CR performance ( Durning, 2011; Schmidt, 2017; Mamede 2016). One study showed that patient appearance did not affect diagnostic accuracy, although physicians processed the case more extensively if the patient appeared ‘rich and clean’ ( Mohamed, 2016).The condition with which the patient presents can have an impact, with complex or atypical presentation of disease leading to a reduction in CR skills ( Durning, 2011; Schmidt, 2017; Harun, 2015; Islam, 2015). One study suggested that the addition of patient factors led to physicians desiring more information, including investigations ( Durning, 2011; McBee, 2015). If patient factors, such as distressing behaviours and poor English proficiency, have a negative impact on CR then it suggests that these patients could be at higher risk of misdiagnosis, incorrect treatment and additional investigation.

There are physician factors which influence CR beyond theoretical knowledge. Studies have suggested that many factors can bias physicians and reduce their CR performance; including recent exposure to a disease presenting in a similar way ( Mamede, 2010; Schmidt, 2014). Salient distracting features at the beginning of a complex case reduced diagnostic accuracy ( Mamede, 2014).One study suggested that physicians’ diagnostic accuracy may decrease with age (St-Onge, 2016). Physicians’ emotions were reported to negatively influence CR, with fear of causing harm to the patient and fear of disappointing seniors being identified ( Harun, 2015; Islam, 2015, Tallentire 2011). One study suggested that physicians found it difficult to reject an incorrect diagnostic suggestion ( Van den Berge, 2012a); however another study did not support this finding ( Van den Berge, 2012b). Some factors can also positively influence a physician’s CR, including higher experience level ( Monajemi, 2012; Monteiro, 2015), the use of checklists ( Sibbald, 2013) and mnemonics ( Chew, 2016), and reflection (Schmidt, 2017; Sibbald, 2011; Schmidt 2014). If physicians are influenced by recent exposure to similar cases, salient distracting features and incorrect diagnostic suggestion, then work to aid physicians’ recognition of these biasing factors could help mitigate against their effect. Experience seems to have a positive influence on CR, however, one study suggested that older physicians have reduced diagnostic accuracy. If this is the case older physicians may have to implement additional methods to maintain their CR abilities. The use of reflection, checklist and mnemonics seem to have a positive influence on CR and implementing these strategies in clinical practice could improve CR.

Some studies suggested that time pressure has a negative effect on CR (Alqahtani, 2014; Durning, 2012), while other studies showed a neutral impact (Alqahtani, 2014; Durning, 2012), this area would benefit from further research. Working in unfamiliar surroundings and a rapidly changing situation were identified as having a negative effect on CR ( Balla, 2012; Tallentire, 2011). This suggests that physicians in new departments or in settings where situations change rapidly may require additional support.

Lastly some studies have tried to assess the impact of more than one contextual factor upon CR and have suggested that this leads to a reduction in CR performance ( Durning, 2011; Durning, 2012),although one study did not show this consistently ( Ratcliffe, 2017), and that this may also lead to an increased desire for information ( McBee, 2015; Durning, 2012).

## Conclusion

In this original review article there is a strong theme emerging from the literature that contexts - patient, physician and environment - affect physician’s CR. In certain patient groups CR may be more challenging and so those groups are potentially vulnerable to poorer care. Physicians may be vulnerable to bias from recent experiences and exposures and their own emotional state. Reflection techniques may help to mitigate against this. The effect of time on CR has shown conflicting results and would benefit from further research. Rapidly changing and unfamiliar surroundings have been identified by physicians as having a negative effect on CR suggesting that CR may be more challenging in certain placements. Finally, combinations of the factors may interact, adding cognitive load on physicians, reducing CR performance and leading to the use of additional health resources.

This area needs further research to assess the impact of these contexts in a real-world setting. There was a lack of research in the literature looking at whether there is variability amongst specialities or locations; it may be that certain specialities or working environments are more supportive for CR.

Medical institutions wish to produce physicians with good CR, improving care for patients and minimising medical error. Our review shows that contexts can influence physicians’ CR, suggesting that altering the context can enhance physicians’ CR. This must be considered in teaching and assessing CR, rather than simply focussing on the individual physician.

It is clear that many factors influence physicians’ CR, and that it is likely that these factors both interact and change from moment to moment. What the literature does suggest is that focusing solely the individual physician is too simplistic and that we need to recognise the interplay between patient, physician and environment.

## Take Home Messages

• Clinical reasoning abilities can vary with the context
• Contexts studied in the literature tend to fall into 3 categories; patient factors, physician factors and environmental factors
• Future teaching and assessment of clinical reasoning needs to consider the context rather than solely focussing on the individual physician

## Notes On Contributors

Alice Mason is a rheumatology trainee in the Wessex region currently working at University Hospital Southampton, UK. She has a strong interest in medical education having spent time as a Wessex education fellow and has been involved in curriculum development for Southampton medical school as well as being a medicine in practice tutor for medical students.

Rachel Locke is Senior Lecturer in International Development (Global Health) at the University of Winchester. She is currently leading the provision of teaching and learning for both undergraduates and postgraduates in global health. Rachel works in partnership with a network of commissioners, professionals and researchers in health to develop programmes of practice-engaged research of national and international significance and consequence.

Rosie M Lusznat is a Psychiatrist and Clinical Psychologist by background and was a Consultant in Old Age Psychiatry, but has now retired from NHS clinical practice. During her Consultant career she was a clinical and educational supervisor; Royal College Tutor; Training Programme Director for Psychiatry; Locality Lead Clinician and Medical Director. Rosie was Associate Dean for Wessex Postgraduate Medical Education from 1999 to 2017. Her current professional roles include Associate Member of the General Medical Council and free-lance educator and researcher. She holds a MA in Education (University of Winchester) and a ILM Certificate in Executive Coaching.

Colin Coles has worked in medical education for nearly fifty years, initially with Southampton Medical School, creating a medical education unit there. He is currently Visiting Professor at the University of Winchester in the Centre for Global Health, which he co-founded. His main academic interest is the education of professional practitioners.

Mike G Masding is the Lead Foundation School Director in England and Co-chair of the UK Foundation Programme Office, and chairs the UK Foundation School Directors Committee. Workplace supervision of Foundation doctors was the subject of his MA(Ed) dissertation at the University of Winchester in 2010. He also continues his clinical work as a Consultant Diabetologist in Poole, Dorset.

## Declarations

The author has declared that there are no conflicts of interest.

## Ethics Statement

Review of literature only and so no ethical approval required. This research did not require Ethics Board approval because it does not involve human or animal subjects.

## External Funding

This article has not had any External Funding

## References

[ref1] ALQahtani D. A. Rotgans J. I. Mamede S. and ALAlwan I. (2016). Does Time Pressure Have a Negative Effect on Diagnostic Accuracy? Academic Medicine. 91(5), pp.710–716. 10.1097/ACM.0000000000001098 26826069

[ref2] Balla J. Heneghan C. Thompson M. and Balla M. (2012). Clinical decision making in a high-risk primary care environment: a qualitative study in the UK. BMJ Open. 2(1), p. e000414. 10.1136/bmjopen-2011-000414 PMC3330259 22318661

[ref3] Chew K. Durning S. and van Merriënboer J. (2016). Teaching metacognition in clinical decision-making using a novel mnemonic checklist: an exploratory study. Singapore Medical Journal. 57(12), pp.694–700. 10.11622/smedj.2016015 26778635 PMC5165179

[ref4] Durning S. Artino A. Pangaro L. van der Vleuten C. (2010). Perspective: Redefining Context in the Clinical Encounter: Implications for Research and Training in Medical Education. Academic Medicine. 85(5), pp.894–901. 10.1097/ACM.0b013e3181d7427c 20520047

[ref5] Durning S. Artino A. Pangaro L. van der Vleuten C. (2011). Context and clinical reasoning: understanding the perspective of the expert’s voice. Medical Education. 45(9), pp.927–938. 10.1111/j.1365-2923.2011.04053.x 21848721

[ref6] Durning S. J. Artino A. R. Boulet J. R. Dorrance K. (2011). The impact of selected contextual factors on experts’ clinical reasoning performance (does context impact clinical reasoning performance in experts?). Advances in Health Sciences Education. 17(1), pp.65–79. 10.1007/s10459-011-9294-3 21505841

[ref7] Eva K. (2003). On the generality of specificity. Medical Education. 37(7), pp.587–588. 10.1046/j.1365-2923.2003.01563.x 12834414

[ref8] Eva K. (2005). What every teacher needs to know about clinical reasoning. Medical Education. 39(1), pp.98–106. 10.1111/j.1365-2929.2004.01972.x 15612906

[ref9] Gmcuk.org. (2019). gmc-uk.org. [online] Available at: https://www.gmc-uk.org/ethical-guidance/ethical-guidance-for-doctors/good-medical-practice(Accessed: 17 September 2019).

[ref10] Guerrasio J. and Aagaard E. (2014). Methods and Outcomes for the Remediation of Clinical Reasoning. Journal of General Internal Medicine. 29(12), pp.1607–1614. 10.1007/s11606-014-2955-1 25092006 PMC4242871

[ref11] Harun N.A. Finlay A. Y. Salek M. S. and Piguet V. (2015). Appropriate and inappropriate influences on outpatient discharge decision making in dermatology: a prospective qualitative study. British Journal of Dermatology. 173(3), pp.720–730. 10.1111/bjd.13946 26076194

[ref12] Higgs J. and Jones M. (2000). Clinical reasoning in the health professions. Oxford: Butterworth-Heinemann.

[ref13] Islam R. Weir C. R. Jones M. Del Fiol G. (2015). Understanding complex clinical reasoning in infectious diseases for improving clinical decision support design. BMC Medical Informatics and Decision Making. 15(1). 10.1186/s12911-015-0221-z PMC4665869 26620881

[ref14] Kohn L. (2009). To err is human. Washington, DC: National Academy Press.

[ref15] Kuhn G. (2002). Diagnostic Errors. Academic Emergency Medicine. 9(7), pp.740–750. 10.1197/aemj.9.7.740 12093717

[ref16] Mamede S. Van Gog T. Schuit S. C. E. Van den Berge K. (2016). Why patients’ disruptive behaviours impair diagnostic reasoning: a randomised experiment. BMJ Quality & Safety. [online] 26(1), pp.13–18. 10.1136/bmjqs-2015-005065 26951796

[ref17] Mamede S. van Gog T. van den Berge K. van Saase J. L. C. M. (2014). Why Do Doctors Make Mistakes? A Study of the Role of Salient Distracting Clinical Features. Academic Medicine. 89(1), pp.114–120. 10.1097/ACM.0000000000000077 24280846

[ref18] Mamede S. van Gog T. van den Berge K. Rikers R. M. J. P. (2010). Effect of Availability Bias and Reflective Reasoning on Diagnostic Accuracy Among Internal Medicine Residents. JAMA. 304(11), p.1198. 10.1001/jama.2010.1276 20841533

[ref19] McBee E. Ratcliffe T. Picho K. Artino A. (2015). Consequences of contextual factors on clinical reasoning in resident physicians. Advances in Health Sciences Education. 20(5), pp.1225–1236. 10.1007/s10459-015-9597-x 25753295

[ref20] Mohamed F. Mamede S. Mohamedani M. Alwan I. A. (2016). The Effect of Patients’ Appearance on Doctors’ Diagnostic Decision Making: Do Poor People Get Poorer Medical Care? Health Professions Education. 2(1), pp.18–23. 10.1016/j.hpe.2016.01.011

[ref21] Monajemi A. Schmidt H.G. and Rikers R. M. J. P. (2012). Assessing Patient Management Plans of Doctors and Medical Students: An Illness Script Perspective. Journal of Continuing Education in the Health Professions. 32(1), pp.4–9. 10.1002/chp.21118 22447706

[ref22] Monteiro S. D Sherbino J. D. Ilgen J. S. Dore (2015). Disrupting diagnostic reasoning: Do interruptions, instructions, and experience affect the diagnostic accuracy and response time of residents and emergency physicians? Academic Medicine. 10.1097/ACM.0000000000000614 25565260

[ref23] Norman G. Sherbino J. Dore K. Wood T. (2014). The Etiology of Diagnostic Errors. Academic Medicine. 89(2), pp.277–284. 10.1097/ACM.0000000000000105 24362377

[ref24] Ratcliffe T. McBee E. Schuwirth L. Picho K. (2017). Exploring Implications of Context Specificity and Cognitive Load in Residents. MedEdPublish. 6(1). https://doi.org/10.15694/mep.2017.000048

[ref25] Schmidt H. van Gog T., C. E. Schuit S. Van den Berge K. (2016). Do patients’ disruptive behaviours influence the accuracy of a doctor’s diagnosis?’ A randomised experiment: Table 1. BMJ Quality & Safety. 26(1), pp.19–23. 10.1136/bmjqs-2015-004109 26951795

[ref26] Schmidt H. G. Mamede S. van den Berge K. van Gog T. (2014). Exposure to Media Information About a Disease Can Cause Doctors to Misdiagnose Similar-Looking Clinical Cases. Academic Medicine. 89(2), pp.285–291. 10.1097/ACM.0000000000000107 24362387

[ref27] Scott I. (2009). Errors in clinical reasoning: causes and remedial strategies. BMJ. 338(jun08 2), pp.b1860–b1860. 10.1136/bmj.b1860 19505957

[ref28] Sibbald M. and Cavalcanti R. B. (2011). The biasing effect of clinical history on physical examination diagnostic accuracy. Medical Education. 45(8), pp.827–834. 10.1111/j.1365-2923.2011.03997.x 21752079

[ref29] Sibbald M. de Bruin A. B. H. Cavalcanti R. B. and van Merrienboer J. J. G. (2013). Do you have to re-examine to reconsider your diagnosis? Checklists and cardiac exam. BMJ Quality & Safety. 22(4), pp.333–338. 10.1136/bmjqs-2012-001537 23386730

[ref30] St-Onge C. Landry M. Xhignesse M. Voyer G. (2015). Age-related decline and diagnostic performance of more and less prevalent clinical cases. Advances in Health Sciences Education. 21(3), pp.561–570. 10.1007/s10459-015-9651-8 26584578

[ref31] Tallentire V. R. Smith S. E. Skinner J. and Cameron H. S. (2011). Understanding the behaviour of newly qualified doctors in acute care contexts. Medical Education. 45(10), pp.995–1005. 10.1111/j.1365-2923.2011.04024.x 21916939

[ref32] Thompson C. (2004). Nurses, information use, and clinical decision making--the real world potential for evidence-based decisions in nursing. Evidence-Based Nursing. 7(3), pp.68–72. 10.1136/ebn.7.3.68 15252900

[ref33] Van den Berge K. Mamede S. van Gog T. Romijn J. A. (2012). Accepting Diagnostic Suggestions by Residents: A Potential Cause of Diagnostic Error in Medicine. Teaching and Learning in Medicine. 24(2), pp.149–154. 10.1080/10401334.2012.664970 22490096

[ref34] Van den Berge Mamede S Van Gog T Saase J (2012). Consistency in diagnostic suggestions does not influence the tendency to accept them. Canadian Medical Education Journal. PMC4563636 26451191

