# Supplementary material for: How Do Contexts Affect Physicians' Clinical Reasoning? A Narrative Review
Source: MedEdPublish (2016). 2020 Feb 14;9:32. [Version 1] doi: 10.15694/mep.2020.000032.1 (PMC10697548; doi:10.15694/mep.2020.000032.1)
Supplement: Peer review history

# Reviewer report: Reviewer response for version 1

**DOI:** 10.21956/mep.19955.r30314

**Published:** 2020-11-28

**Authors:** Mahdi Movahed-Abtahi

This review has been migrated. The reviewer awarded 5 stars out of 5 An Excellent Work! The authors correctly and strongly mentioned "the fusion of horizons" as noted by a great hermenutist. Reflex-thinking and Hermeneutics are two main intuitional skills that emprove other educational skills in medical students and physicians. From such perspective, my sugestions for revising the Figure 2 are as following:1- Doctor factors play role in the centre of drawn system (because of reciever`s interpretation)2- Patient narration play roles as sending messages while doctor interpretation play roles as recieving messages.3- Enviromental factors are surrounding both doctor and patient factors.4- Get please inspired from "the thoery of systems"5- I suggest drowing two interpretational clouds that may fuse, contact, or penetrat into each other, surrounded by enviromental factors:a) Every patient or doctor represent herself/himself through narrative or interpretive approaches, respectively.b) The best diagnosis will happen while two horizions of world (patient narration and doctor interpretation) fuse.c) More exploration will happen if doctor`s horizon penetrates patient`s world.d) Doctor`s decision may affected if patient`s horizon penetrates doctor`s world.6- The "world" includes the inside enviroment and world view of patient or doctor.

Reviewer Expertise:

NA

No decision status is available

# Reviewer report: Reviewer response for version 1

**DOI:** 10.21956/mep.19955.r30316

**Published:** 2020-02-17

**Authors:** Felix Silwimba

**Affiliations:** 1 University of Lusaka

This review has been migrated. The reviewer awarded 5 stars out of 5 this study is important and informative . it clearly informs the context to consider in teaching clinical reasoning to clinicians. I recommend to all medical educators.

Reviewer Expertise:

NA

No decision status is available

# Reviewer report: Reviewer response for version 1

**DOI:** 10.21956/mep.19955.r30313

**Published:** 2020-02-14

**Authors:** Leila Niemi-Murola

**Affiliations:** 1 University of Helsinki

This review has been migrated. The reviewer awarded 4 stars out of 5 This is an interesting article focusing on a clinically highly relevant topic, clinical reasoning skills. It is a skill very challenging to teach and the authors illustrate the factors causing this difficulty. Clinical reasoning depends on the context, and it is a skill sensitive to cognitive overload.The search strategy and results have been described adequately. Picture 2 is very illustrative. The authors describe the three categories and the effects in detail. This is a very useful article for clinicians coaching the residents in busy clinical settings and it is also recommended for teachers using emergency simulations as a learning method.

Reviewer Expertise:

NA

No decision status is available

# Reviewer report: Reviewer response for version 1

**DOI:** 10.21956/mep.19955.r30315

**Published:** 2020-02-14

**Authors:** Balakrishnan Nair

**Affiliations:** 1 Centre for Medical Professional Development and University of Newcastle

This review has been migrated. The reviewer awarded 4 stars out of 5 This is a very interesting topic and this study is very useful for clinicians and teachers . Diagnostic error happens because of the contexts, the authors have described . It is interesting to see positive impact of reflexion. A study after understanding the impact of context on clinical reasoning and diagnostic accuracy will be worthwhile to do.I will try to improve my diagnostic accuracy reflecting on this paper . These are good teaching points too.

Reviewer Expertise:

NA

No decision status is available
